# Perspectives Regarding the Role of Biochanin A in Humans

**DOI:** 10.3389/fphar.2019.00793

**Published:** 2019-07-12

**Authors:** Chen Yu, Peng Zhang, Lixin Lou, Yang Wang

**Affiliations:** ^1^Department of Infectious Diseases, First Hospital of Jilin University, Changchun, China; ^2^Department of Pediatrics, University of Oklahoma Health Sciences Center, Oklahoma City, OK, United States

**Keywords:** biochanin A, chemopreventive, inflammation, neuroprotective effect, bioavailability

## Abstract

Biochanin A (BCA) is an isoflavone mainly found in red clover with poor solubility and oral absorption that is known to have various effects, including anti-inflammatory, estrogen-like, and glucose and lipid metabolism modulatory activity, as well as cancer preventive, neuroprotective, and drug interaction effects. BCA is already commercially available and is among the main ingredients in many types of supplements used to alleviate postmenopausal symptoms in women. The activity of BCA has not been adequately evaluated in humans. However, the results of many *in vitro* and *in vivo* studies investigating the potential health benefits of BCA are available, and the complex mechanisms by which BCA modulates transcription, apoptosis, metabolism, and immune responses have been revealed. Many efforts have been exerted to improve the poor bioavailability of BCA, and very promising results have been reported. This review focuses on the major effects of BCA and its possible molecular targets, potential uses, and limitations in health maintenance and treatment.

## Introduction

Phytoestrogens are compounds found in plants with a molecular structure and size resembling those of estrogens. Plant flavonoid isoflavones are the most popular among the many estrogenic compounds (Heinonen et al., 1999). In humans, the main dietary sources of isoflavones are soybean and soybean products. When these types of food are consumed, they have multiple effects (Vitale et al., 2013). Epidemiological studies have indicated that populations with a high isoflavone intake through soy consumption have lower rates of several cancers, such as breast, prostate, bladder, gastric, and colon cancer (Kweon et al., 2013; Zhang et al., 2017; Perez-Cornago et al., 2018; Wada et al., 2018; You et al., 2018; Grainger et al., 2019). Isoflavones are considered chemoprotective and can be used as an alternative therapy for a wide range of hormonal disorders (van Duursen, 2017; Křížová et al., 2019).

**Figure 1:**
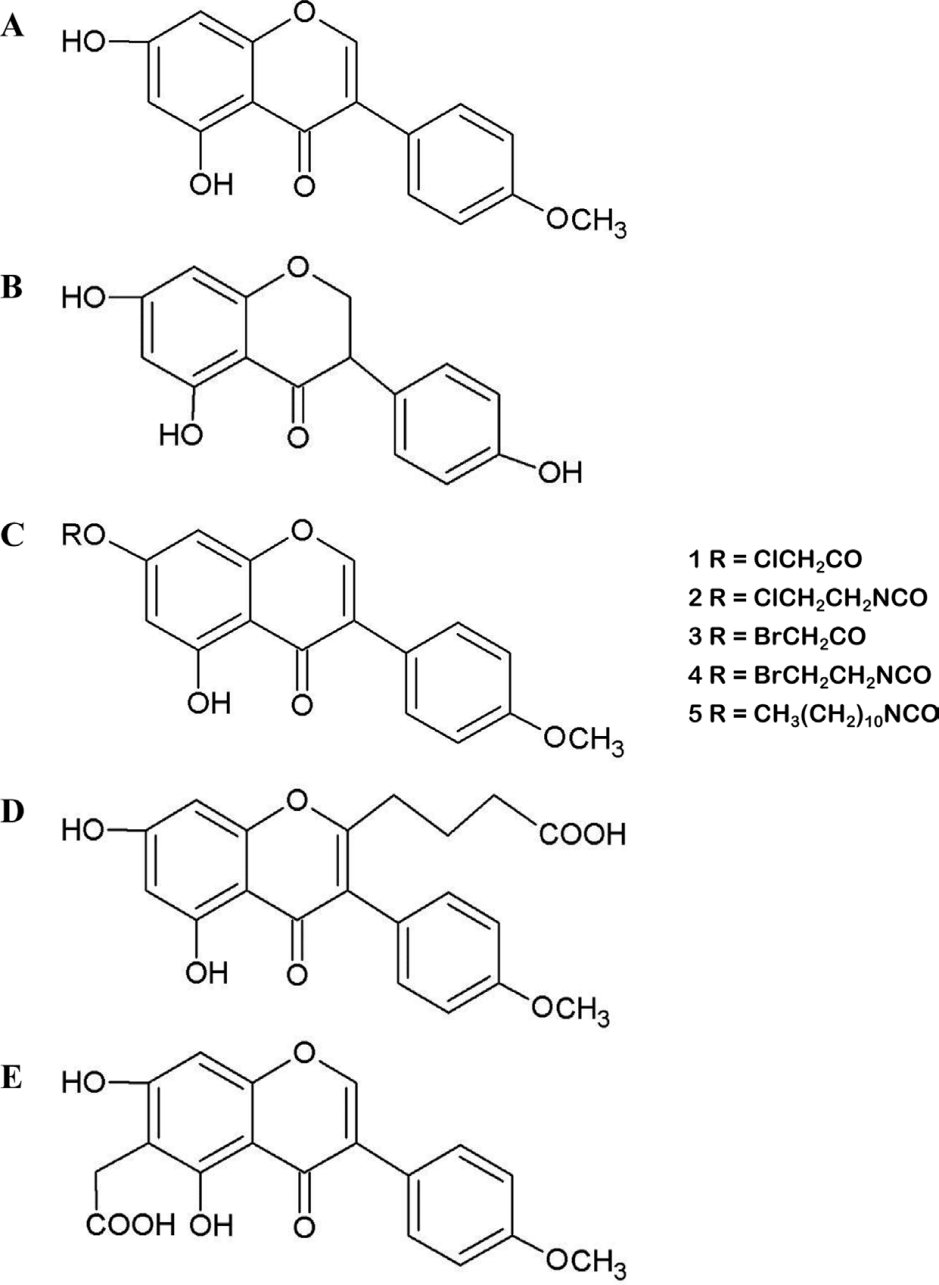
**(A)** Molecular structure of biochanin A (BCA). **(B)** Molecular structure of genistein (GEN). **(C)** Structures of synthesized esters (1, 3) and carbamate esters (2, 4, 5), which are BCA derivatives. **(D** and **E)** Molecular structures of carboxyalkyl BCA.

Biochanin A (5,7-dihydroxy-4’-methoxy-isoflavone, BCA) (**Figure 1A**) is an isoflavone present in red clover, cabbage, alfalfa, and many other herbal products (Cassady et al., 1988). BCA may occur as an aglycon and can also be used as a hormone alternative therapy. BCA plays complex roles in the regulation of multiple biological functions by binding DNA and some specific proteins or acting as a competitive substrate for some enzymes (Roberts et al., 2004; Křížová et al., 2019; Liang et al., 2019; Luo et al., 2019). BCA is the methylated precursor of the isoflavone genistein (GEN), which is another well-studied isoflavone. In the gut, intestinal bacteria convert BCA to its demethylated form (Setchell et al., 2001). However, the biological effects of BCA observed *in vitro* and *in vivo* are not identical to those of GEN. Recently, medical research focusing on BCA has increased because of its various purported biological activities, including its antioxidant, anti-inflammatory, anti-infective, and anticarcinogenic effects, and BCA has been used for several purposes, such as to treat estrogen deficiency and pain and reduce the severity of nerve damage (Puli et al., 2006; Medjakovic and Jungbauer, 2008). This extract from plants is already commercially available because of its potential benefits to human health and because it is considered innocuous (Howes et al., 2002; Atkinson et al., 2004; Beck et al., 2005; Sklenickova et al., 2010). Most commercial products are composed of several isoflavone contents, including BCA (Booth et al., 2006; Ahmad et al., 2013). These botanical dietary supplements are sold in tablet form in several countries and are commonly used to alleviate postmenopausal symptoms in women. The use of these products is clearly increasing. However, BCA is a Biopharmaceutics Classification System Class II drug because of its poor water solubility. Given that studies are increasingly focusing on the effects of BCA (**Table S1**), it is timely and appropriate to obtain in-depth knowledge of the effects of BCA and critically evaluate the paradoxical observations in the published literature.

## BCA Has Chemopreventive Activity Against Various Cancers

**Figure 2:**
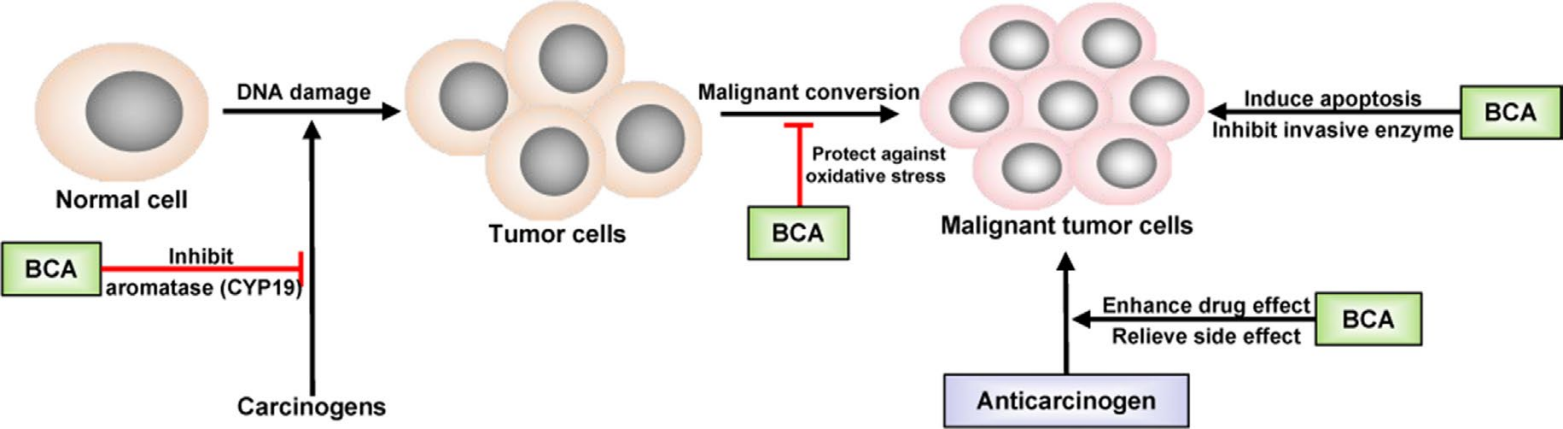
Schematic of the anticancer effect of BCA. →, direct stimulation; ⊥, direct inhibition.

Inspired by epidemiological evidence suggesting that a relationship exists between the consumption of certain foods containing isoflavones and decreased cancer incidence in humans, BCA has been evaluated in many studies related to cancer treatment. The first study was performed in 1988 in hamster embryo cell cultures and found that BCA inhibited carcinogen activation (Cassady et al., 1988). Subsequently, studies investigating the anticancer activity of BCA were carried out in different cancer cell lines, followed by animal models. Many types of tumors could be inhibited by BCA, such as lung cancer (Lee et al., 1991), prostate cancer (Peterson and Barnes, 1993; Sun et al., 1998), gastrointestinal tract cancer (Yanagihara et al., 1993), pancreatic cancer (Bhardwaj et al., 2014), breast cancer (Balabhadrapathruni et al., 2000; Sehdev et al., 2009), osteosarcoma (Hsu et al., 2018; Zhao et al., 2018), malignant melanoma (Xiao et al., 2017), and tumors of the central nervous system (Sehm et al., 2014). However, the ability of BCA to inhibit the growth of some types of cancer cells was weaker than that of GEN (Peterson and Barnes, 1991), but the anticancer usage of BCA might be broader because of its targeting of anticancer activity, especially in malignant brain tumors (Sehm et al., 2014). BCA is a potent inhibitor of cytochrome P450 (CYP) and, thus, may be useful as a chemopreventive agent against hydrocarbon-induced carcinogenesis, and BCA has an inhibitory effect on the metabolism of some carcinogens, such as benzo(a)pyrene, by binding DNA (Chae et al., 1991; Lee et al., 1991; Lee et al., 1992). BCA significantly reduces the synthesis of prostaglandin E2 and thromboxane B2 and the activity of CYP19/aromatase (Almstrup et al., 2002; Wang et al., 2008), leading to cyclooxygenase-2 (COX-2) inhibition (Lam et al., 2004; Lim et al., 2013). The chronic activation or overexpression of COX-2 has been shown to be correlated with the development of cancer, particularly at sites of inflammation. The inhibition of COX-2 has been linked to the decreased development of some types of cancer (Dannenberg and Subbaramaiah, 2003). BCA provides protection against oxidative stress and inhibits the expression and activity of invasive enzymes (Ullah et al., 2009; Sehdev et al., 2009). In earlier studies, apoptosis was regarded as the major mechanism underlying the antitumor activity of BCA (Yanagihara et al., 1993; Yanagihara et al., 1996; Balabhadrapathruni et al., 2000; Puthli et al., 2013). In recent studies, more details regarding the antitumor effects of BCA have been discovered, such as the signaling pathways and effects on vascular invasion (Xiao et al., 2017; Lai et al., 2018; Hsu et al., 2018). BCA could effectively inhibit the proliferation of lung cancer cells by downregulating Ki-67, induce apoptosis by activating the cleavage of caspase-3 and caspase-9, and suppress cell migration by downregulating matrix metallopeptidase-2 (MMP-2) and vascular endothelial growth factor (VEGF) (Lai et al., 2018; Hsu et al., 2018). BCA inhibited cell migration and invasion in a dose-dependent manner and upregulated the expression of key proteins in the NF-κB and mitogen-activated protein kinase (MAPK) signaling pathways (Xiao et al., 2017). BCA acts as a remarkable pro-oxidant factor, significantly enhancing radiotoxicity in colon cancer cells *in vitro* (Puthli et al., 2013). The anticancer effects of BCA are presented in **Figure 2**. BCA also enhances the effects of some anticarcinogens and relieves their side effects. The most important point is that BCA showed no such effects on normal tissues and cells at the moderate dose at which it inhibited cancer cells (Sehdev et al., 2009; Sehm et al., 2014; Hsu et al., 2018). BCA is considered a potent chemopreventive and/or therapeutic agent against cancer.

## BCA May Play a Therapeutic Role in Metabolic Disorders

BCA is metabolized in the gut to GEN or formononetin, which is converted to daidzein and then to equol (Knight and Eden, 1996). BCA is an estrogen receptor (ER) α and ERβ agonist that promotes transcriptional repression and activation at physiological levels. BCA may act as a natural selective ER modulator that elicits distinct clinical effects from estrogens used for hormone replacement by selectively recruiting coregulatory proteins to ERβ to trigger transcriptional pathways. As a promising alternative estrogen therapy, BCA might be used for the management of the renal and cutaneous changes observed in postmenopausal women while preventing bone loss (An et al., 2001; Beck et al., 2003; Hellström and Muntzing, 2012; Elsherbini et al., 2017; Galal et al., 2018). BCA is well known for its regulation of blood glucose and has significant effects in type 2 diabetes mellitus *in vivo* by affecting mechanisms that influence autophagy, differentiation, inflammation, and metabolism (Mehrabadi et al., 2018; Nikolic et al., 2018; Oza and Kulkarni, 2018). BCA exerts lipid-lowering effects by increasing the cholesterol efflux and preventing cholesterol ester transport (Xue et al., 2017). BCA also has a gastroprotective effect through the enhancement of cellular metabolic cycles, as evidenced by increases in superoxide dismutase (SOD) and nitric oxide (NO) activity, decreases in the malondialdehyde (MDA) and Bax levels, and increases in Hsp70 expression (Hajrezaie et al., 2015). Ovariectomy results in a marked increase in body weight and a decrease in femoral bone mineral density and trabecular bone, which are common findings after 17β-estradiol (E2) treatment. BCA treatment can effectively prevent the ovariectomy-induced increases in bone loss and bone turnover possibly by increasing osteoblast activity and decreasing osteoclast activity. All stages of bone formation, including osteoblast proliferation, differentiation, and mineralization, are influenced by BCA (Su et al., 2013a; Kaczmarczyk-Sedlak et al., 2015; Mohamed et al., 2018). BCA has been reported to stimulate endothelial NO synthase (eNOS) and the release of NO, which is vasodilatory and vasoprotective. BCA has been shown to attenuate hypertension in ovariectomized rats by decreasing the systolic, diastolic, and mean arterial blood pressures; decreasing oxidative stress and the tumor necrosis factor-α (TNF-α) levels; and increasing the NO levels in an eNOS-dependent manner (Sachdeva et al., 2016). BCA regulates bone formation by preventing adipogenesis and enhancing osteoblast differentiation in mesenchymal stem cells and has beneficial regulatory effects on bone formation. BCA may be a useful agent in the treatment and prevention of osteoarthritis (Su et al., 2013b; Wu et al., 2014). BCA is well known for its antidiabetic and hypolipidemic effects. Its hypolipidemic effect in diabetes is achieved at least partially by the activation of hepatic peroxisome proliferator-activated receptor α (PPARα) (Qiu et al., 2012). BCA increases the circulating insulin levels and improves insulin sensitivity, leading to body weight control, an increase in liver glycogen, and a decrease in plasma glucose (Harini et al., 2012; Oza and Kulkarni, 2018). BCA also has protective effects on β cells in diabetic rats (Azizi et al., 2014). BCA ameliorates hepatic steatosis and insulin resistance by modulating lipid and glucose metabolism in obese rats (Park et al., 2016). Moreover, BCA helps prevent diabetic complications because it is an excellent inhibitor of insulin and hemoglobin glycosylation and has anti-inflammatory activity (Asgary et al., 2002; Chundi et al., 2016; Patil et al., 2016; Mehrabadi et al., 2018). BCA inhibits fatty acid amide hydrolase and may be used as a novel analgesic agent (Thors et al., 2010). BCA has been shown to inhibit melanogenesis *in vitro* and *in vivo* because of its tyrosinase inhibitory effect and could be a promising candidate as a skin-whitening agent for the treatment of skin hyperpigmentation disorders (Lin et al., 2011). Therefore, BCA may have wide application prospects in the treatment of metabolic diseases.

## BCA Affects Proinflammatory Responses

**Figure 3:**
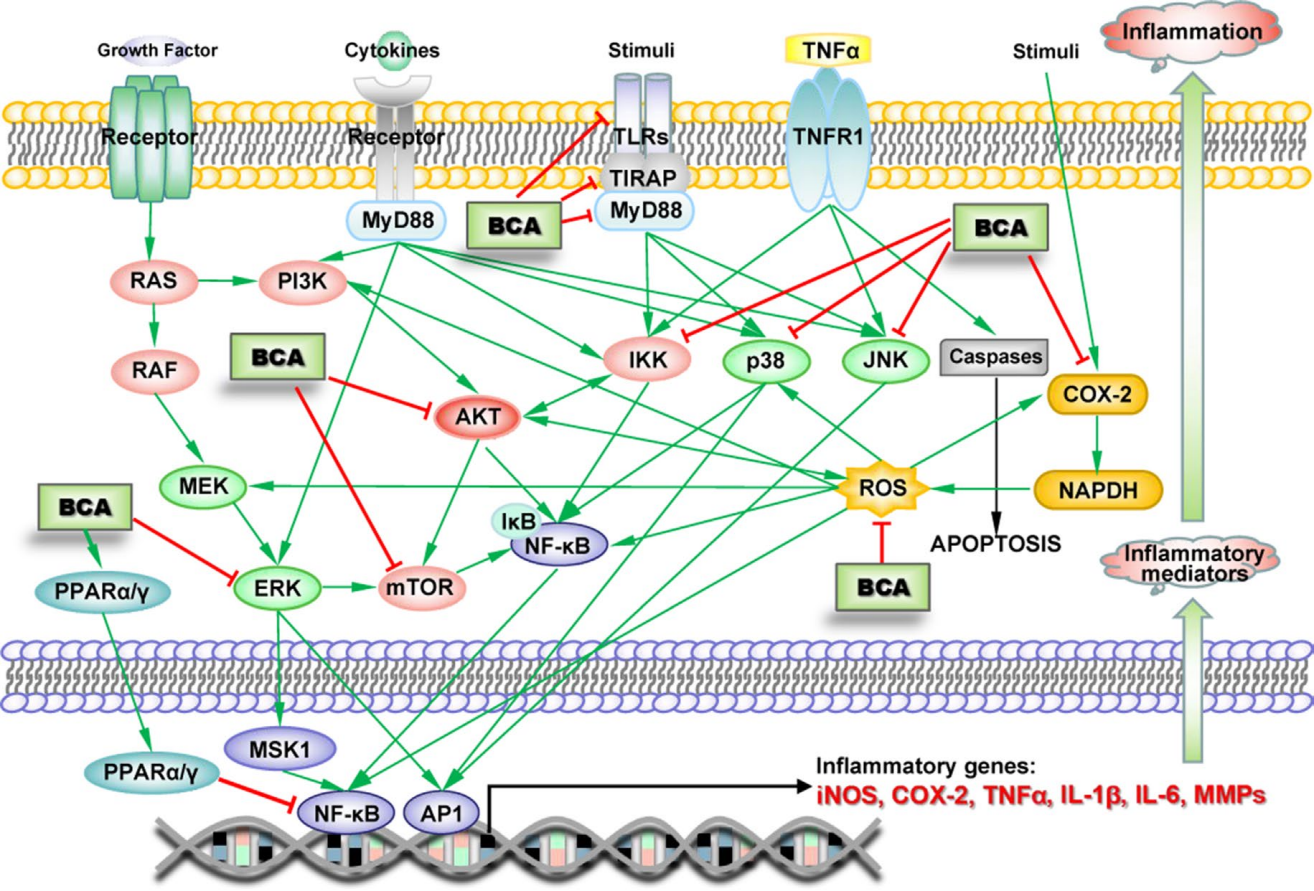
Schematic of the BCA targets (proteins and genes) in key inflammation-associated signaling pathways. →, direct stimulation; ⊥, direct inhibition.

Numerous studies have indicated the anti-inflammatory effects of BCA, which were first demonstrated in microglia in 2007, when BCA was shown to inhibit lipopolysaccharide (LPS)-induced activation of microglia (Chen et al., 2007). The anti-inflammatory effect of BCA has been demonstrated in many other types of cells, including macrophages, various cancer cells, and endothelial cells, in numerous *in vivo* experiments (Lee and Choi, 2005; Park et al., 2006; Kole et al., 2011; Ming et al., 2015). BCA inhibits the production of inflammatory mediators, such as TNF-α, interleukin-1β (IL-1β), IL-6, iNOS, COX-2, MMP-9, and NO, in various inflammatory responses and tissue injury by attenuating the ERK-MAPK/MSK1 cascade, inhibiting the TLR/TIRAP/MyD88 pathway, inhibiting IκB kinase (IKK) activity, and activating PPARα as an estrogen at low concentrations or PPARγ by binding PPARγ at high concentrations, leading to the NF-κB-driven inhibition of gene transcription and decreased expression of TNF-α, IL-1β, IL-6, iNOS, COX-2, and MMP-9 (**Figure 3**) (Lee and Choi, 2005; Vanden Berghe et al., 2006; Mueller et al., 2010; Kole et al., 2011; Qiu et al., 2012; Breikaa et al., 2013a; Wang et al., 2015c; Zhang and Chen, 2015; Wu et al., 2018). A study claimed that BCA upregulated the production of IL-4 *via* the activation of the PKC/p38/AP-1 and PI3K/PKC/NF-AT pathways (Park et al., 2006). However, some recent studies drew completely different conclusions, namely, BCA did not increase the production of IL-4 and rather suppressed its increase upon stimulation (Ko et al., 2011; Chung et al., 2013). BCA also inhibits AKT/MAPK (ERK, JNK, and p38)/mTOR activation (Chung et al., 2013; Bhardwaj et al., 2014; Jain et al., 2015); this pathway is involved in the regulation of NF-κB and other transcription factors (such as MSK1 and AP1). Reactive oxygen species (ROS) and COX-2 are important proinflammatory factors that can stimulate transcription factors to increase inflammatory mediator expression. BCA scavenges ROS and increases SOD activity (Xue et al., 2017; Zhao et al., 2018). BCA significantly reduces the synthesis of prostaglandin E2 and/or thromboxane B2 by inhibiting COX-2 expression (Lam et al., 2004; Lim et al., 2013). Several BCA targets exert anti-inflammatory effects on the pathways triggered by different inducers in various types of cells (**Figure 3**). In animal models of acute and chronic inflammation, BCA protects against organ injury by exerting robust anti-inflammatory and antioxidant effects (Ko et al., 2011; Breikaa et al., 2013b; Oh et al., 2016).

BCA could influence many types of diseases associated with inflammation because of its effects on several inflammatory signaling pathways. Further research is needed to obtain an in-depth understanding of its impact on these diseases.

## BCA Influences Pathogen Infection

BCA was found to have an antiviral potential in 1996; BCA inhibited human herpesvirus 6 antigen expression by suppressing the phosphorylation of protein tyrosine kinases (Cirone et al., 1996). BCA also inhibited influenza A nucleoprotein production, reduced virus-induced caspase 3 cleavage and the nuclear export of viral RNP complexes, and enhanced the effects of the neuraminidase inhibitor zanamivir in influenza H5N1 virus-infected lung epithelial cells by affecting signaling pathways to ultimately reduce the virus-induced activation of AKT, ERK½, and NF-κB. BCA also inhibits the virus-induced production of cytokines, such as IL-6, IL-8, TNF-α, and IP-10 (Sithisarn et al., 2013). BCA enhances H5N1-induced ROS formation, whereas antioxidant use suppresses BCA-induced ROS formation and strongly increases its anti-H5N1 activity in H5N1-infected human alveolar basal epithelial cells (Michaelis et al., 2014). However, BCA does not have broad-spectrum antiviral activity, and it has been demonstrated that BCA does not exhibit anti-enterovirus 71 activity (Li et al., 2017).

Some researchers have studied BCA in the context of antibacterial treatment, but most results of treatment with BCA alone have been negative. However, a previous study found that BCA had selective antibacterial action; BCA inhibited all clostridia, which may be responsible for severe intestinal infections, but not bifidobacteria, which are regarded as probiotic microorganisms (Sklenickova et al., 2010). Another study found that BCA has an inhibitory effect on intracellular bacteria belonging to the genus *Chlamydia* and is a potent inhibitor of *Chlamydia* spp. (Hanski et al., 2014). A recent study demonstrated that BCA induced AMPK/ULK1/mTOR-mediated autophagy and macrophage extracellular traps (METs), which enhanced defense against *Salmonella* infection *in vitro* and *in vivo*. In addition, BCA inhibits both inflammatory and anti-inflammatory responses when the body is infected by bacteria. These findings provide basic data regarding the control of infections by enhancing the host immune defense and indicate a potential new strategy to overcoming the desperate scarcity of new therapeutic approaches.

## Neuroprotective Effects of BCA

**Figure 4:**
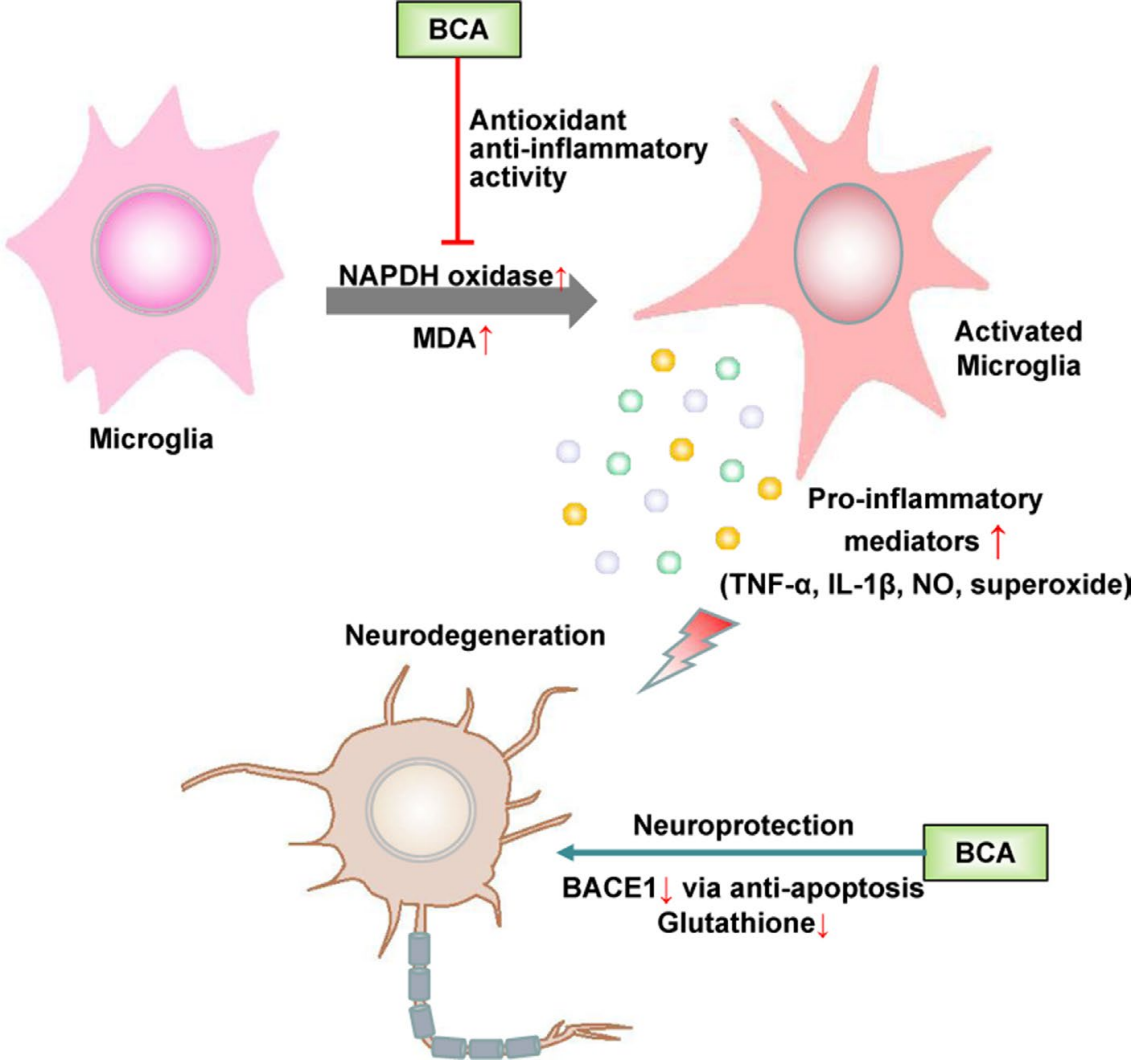
Schematic of the neuroprotective effects of BCA. →, direct stimulation; ⊥, direct inhibition.

Microglia, which are the resident immune cells in the brain, play a role in immune surveillance and host defense against infectious agents under normal conditions. Activated microglia produce a variety of proinflammatory factors, including cytokines, such as TNF-α, and the free radicals NO and superoxide. The accumulation of these factors is deleterious to neurons (Huang et al., 2005). The abnormal activation of microglia is closely associated with some neurodegenerative diseases, such as Parkinson’s disease (PD), Alzheimer’s disease (AD), and frontotemporal dementia (FTD) (Bachiller et al., 2018). Accumulating evidence suggests that estrogen inhibits the LPS-induced inflammatory response in microglia and has a neuroprotective effect (Suuronen et al., 2005; Pozzi et al., 2006; Vegeto et al., 2006). As a promising phytoestrogen, many studies have focused on the effect of BCA on neurodegenerative diseases, especially PD and AD (**Figure 4**).

BCA has been shown to protect dopaminergic neurons against LPS-induced damage by inhibiting the activation of microglia; the generation of proinflammatory factors, such as TNF-α, IL-1β, NO, and superoxide (Chen et al., 2007); and MAPK signaling pathways in microglia (Wu et al., 2015; Wang et al., 2016). BCA inhibits nicotinamide adenine dinucleotide phosphate oxidase (NADPH oxidase) activation and malondialdehyde (MDA) production, thereby increasing SOD and glutathione peroxidase (GPx) activity in the brain. The neuroprotective effect of BCA is partially associated with its antioxidant activity and ability to maintain a redox imbalance (Occhiuto et al., 2009; Wang et al., 2015b; Yu et al., 2017). BCA also exerts a neuroprotective effect against L-glutamate-induced cytotoxicity, which plays a crucial role in neuronal cell death in various neurodegenerative diseases and reduces glutathione levels (Tan et al., 2013; Biradar et al., 2014). BCA is a potent, reversible, and selective oxidase-B (MAO-B) inhibitor because of the hydrophobic interactions between BCA and MAO-B, and MAO-B inhibitors are widely used in the treatment of PD and have potential in the future treatment of AD (Zarmouh et al., 2017). BCA effectively inhibits the activity of beta-site amyloid precursor protein cleaving enzyme 1 (BACE1) not only *via* a mitochondria-dependent apoptosis pathway but also by binding the allosteric site of BACE1; BACE1 accumulation is among the major histological hallmarks of AD (Youn et al., 2016). BCA may be used as a preventative and/or therapeutic agent for AD by binding the preformed fibril structure of β-amyloid_25–35_ and inhibiting β-amyloid_25–35_-induced apoptosis by suppressing caspase activity (Ghobeh et al., 2014; Tan and Kim, 2016). Furthermore, BCA has been shown to have neuroprotective effects in cerebral ischemia/reperfusion and subarachnoid hemorrhage based on the inhibition of inflammatory injury and neuronal apoptosis and the induction of glutamate oxaloacetate transaminase-mediated glutamate metabolism (Wang et al., 2015c; Khanna et al., 2017; Wu et al., 2018).

## BCA Plays Complex Roles in Paradoxical Drug–Drug Interactions

Multidrug resistance (MDR) is a major obstacle to the success of cancer chemotherapy and is a complex and multifactorial phenomenon. One important classical mechanism of MDR is the overexpression of drug efflux transporters, such as P-glycoprotein (P-gp). P-gp confers resistance by actively pumping cytotoxic drugs out of cancer cells (Savas et al., 1992). As a multidrug transporter, P-gp also influences the distribution of many other types of drugs (Singh et al., 2012; Stępień et al., 2012). However, BCA can inhibit P-gp-mediated cellular efflux by modulating P-gp ATPase activity without changing the cellular P-gp level (Zhang and Morris, 2003; Zhang et al., 2010; Chung et al., 2005; Dash and Konkimalla, 2017). Interestingly, BCA has been found to stimulate P-gp in some studies (An and Morris, 2010). Therefore, the effect of BCA on P-gp may be substrate dependent. BCA differentially affects the oral bioavailability of some P-gp substrates (Peng et al., 2006; An and Morris, 2010; Singh et al., 2012; Li et al., 2016). BCA can also inhibit non-P-gp-mediated pathways in MDR (Versantvoort et al., 1993), such as MDR-associated protein 1 (MRP1)-mediated drug transport (Versantvoort et al., 1993; Nguyen et al., 2003) and breast cancer resistance protein (BCRP)-mediated cellular efflux, because BCA sulfate is a substrate of BCRP (An and Morris, 2010). Oatp3 is a highly expressed influx/efflux transporter in the rat small intestine that plays an important role in limiting the absorption and, therefore, bioavailability of its substrates. BCA has been shown to inhibit Oatp3, causing a decrease in drug bioavailability (Peng et al., 2006). BCA synergizes with quinolones to inhibit *Staphylococcus aureus* by increasing the accumulation of ciprofloxacin and suppressing the bacterial expression of the norA protein and the efflux system [adenosine triphosphate (ATP)-binding ABC transporters] but has no inhibitory effect on the bacteria alone (Liu et al., 2011; Zou et al., 2014). Synergy between quinolones and BCA has also been observed in the treatment of pathogenic mycoplasma and *Mycobacterium avium* (Jin et al., 2017; Cannalire et al., 2017). The most common mechanism underlying these drug–drug interactions is the inhibition of the CYP system, which is responsible for the metabolism of nearly 90% of drugs in humans. BCA exerts minimal effects on CYP isoforms other than CYP1A2 and CYP3A4. The consumption of BCA along with other drugs is assumed to be safe with a minimal possibility of alterations in the pharmacokinetics of the coadministered drugs (Arora et al., 2015; Kopečná-Zapletalová et al., 2017). However, BCA was found to enhance the distribution and cytotoxicity of some drugs *in vivo* and cause unwanted pharmacokinetic interactions (Zhang and Morris, 2003; An and Morris, 2010; Li et al., 2016). BCA ameliorated the adverse effects of some anticarcinogens by increasing their cellular uptake and efficacy to reverse drug resistance, significantly improving serum oxidant/antioxidant activity or modulating the proliferation and apoptosis of cancer cells (Youssef et al., 2016; Galal et al., 2018). BCA acts as a nephroprotective agent in the presence of certain chemotherapeutics, such as cisplatin, because of its anti-inflammatory and antiapoptotic activities (Suliman et al., 2018) and protects heart tissue and the kidney against arsenic toxicity because of its antioxidant characteristics (Jalaludeen et al., 2015, Jalaludeen et al., 2016). The complex roles of BCA in paradoxical drug–drug interactions are summarized in **Table S2**.

It is possible that BCA could be used alone or in combination with other drugs to reverse MDR. However, the probability of pharmacokinetic interactions must be carefully considered before the coadministration of BCA with other drugs.

## Bioavailability of BCA

Because of its potential benefits, BCA has been studied in many *in vitro* and *in vivo* experiments. However, BCA is a poorly soluble bioflavonoid, and this characteristic prevents its oral absorption. BCA has a high clearance and a large apparent volume of distribution, and its bioavailability is poor. BCA (**Figure 1A**) has been reported to undergo extensive metabolism *in vivo*; GEN (**Figure 1B**) and sulfate and glucuronide conjugates are the major metabolites in the blood of humans. Significant levels of BCA and GEN conjugates were detected in plasma and bile *in vivo* (Moon et al., 2006). These metabolites may contribute to the chemopreventive effects of BCA and might have longer exposure periods depending on enterohepatic recycling. The administration of multiple flavonoids, including BCA, leads to increased flavonoid bioavailability and decreased clearance potentially caused by increased enterohepatic cycling (Moon and Morris, 2007).

However, the low biological availability and poor aqueous solubility of BCA limit its usefulness as a chemotherapeutic agent. Various attempts have been made to improve the solubility and bioavailability of BCA, including the use of liposomes (Hendrich et al., 2002), dispersion agents (Han et al., 2011), silver nanoparticles (Sekine et al., 2011), different film formulations for buccal delivery (Hanski et al., 2014), nanostructured lipid carriers (Wang et al., 2013), nanostructured lipid carriers modified with polyethylene glycol (PEG) (Wang et al., 2015a), enteric-coated microparticles (Sachdeva et al., 2016), micelles (Wu et al., 2017), and inclusion complexes with cyclodextrins (Nikolic et al., 2018). Ester and carbamate ester derivatives of BCA (**Figure 1C**) and several carboxy–BCA compounds (**Figures 1D, E**) have been synthesized. These derivatives maintain estrogenic and cancer chemopreventive activities, and some have better metabolic stability than BCA in cells (Somjen et al., 2005; Kohen et al., 2007; Somjen et al., 2011; Fokialakis et al., 2012). These efforts have enhanced the solubility and bioavailability of BCA while maintaining its efficacy and activity. These findings provide excellent prospects for the application of BCA in the treatment of various diseases.

## Conclusion

BCA has shown many potential benefits in numerous *in vitro* and *in vivo* studies. However, the safety of supplements containing BCA is unknown, and actual evidence from patients is limited; therefore, more research needs to be performed in this field.

## Author Contributions

YW conceived the general idea. CY, LL, and PZ wrote the first draft. YW revised the manuscript. All authors read and approved the final manuscript.

## Funding

This study was supported by the National Natural Science Foundation of China (Grant Number 81801972).

## Conflict of Interest Statement

The authors declare that the research was conducted in the absence of any commercial or financial relationships that could be construed as potential conflicts of interest.
