# Supplementary material for: Perspectives Regarding the Role of Biochanin A in Humans
Source: Front Pharmacol. 2019 Jul 12;10:793. doi: 10.3389/fphar.2019.00793 (PMC6639423; doi:10.3389/fphar.2019.00793)
Supplement: Supplementary file 1 [file Table_1.docx]

| **Table S1 Pharmacological activity of BCA and related references** | | | | |
| --- | --- | --- | --- | --- |
| **Pharmacology** | | **Mechanism** | ***In vivo* or *in vitro*** | **References** |
| Anti-cancer activity | Hydrocarbon-induced carcinogenesis | Inhibiting P-450 induced by β-naphthoflavone | *in vitro* and *in vivo* | Chae et al., 1991 |
|  | Prostate cancer | Via the EGF receptor‐mediated signaling transduction cascade | *in vitro* | Peterson et al., 1993 |
|  | Gastrointestinal tract cancer | Activating a signal transduction pathway responsible for apoptosis | *in vitro* and *in vivo* | Yanagihara et al., 1993 |
|  | Gastric cancer | Inducing G2 arrest and cell cycle block via the p53-mediated DNA damage response | *in vitro* | Yanagihara et al., 1996 |
|  | Prostate cancer | Increasing UDP-glucuronosyltransferase activity and decreasing prostate specific antigen production | *in vitro* | Sun et al., 1998 |
|  | Breast cancer | Inducing G2/M arrest and apoptosis | *in vitro* | Balabhadrapathruni et al., 2000 |
|  | Breast cancer | Aromatase inhibition | *in vitro* | Almstrup et al., 2002 |
|  | Breast cancer | Inhibiting CYP19 activity and gene expression | *in vitro* | Wang et al., 2008 |
|  | Breast cancer | Selectively targeting cancer cells and inhibiting multiple signaling pathways | *in vitro* | Sehdev et al., 2009 |
|  | Colon tumor | Enhancing the radiotoxicity of colon cancer cells | *in vitro* | Puthli et al., 2013 |
|  | Pancreatic cancer | Inducing apoptosis and inhibiting the activation of Akt and MAPK | *in vitro* | Bhardwaj et al., 2014 |
|  | Malignant brain tumors | Antiedematous and antiangiogenic properties | *in vitro* and *in vivo* | Sehm et al., 2014 |
|  | Gliomas | Inhibiting the activation of proangiogenic proteins (ERK/AKT/mTOR), chemical hypoxia-inducible factor-1α and vascular endothelial growth factor and blood vessel formation | *in vitro* | Jain et al., 2015 |
|  | Malignant melanoma | Inhibiting cell invasion and modulating the NF-κB and MAPK signaling pathways | *in vitro* | Xiao et al., 2017 |
|  | Lung cancer | Suppressing the VEGF/VEGFR2 signaling pathway | *in vitro* and *in vivo* | Lai et al., 2018 |
|  | Osteosarcoma | Inducing apoptosis by activating caspase-3 and decreasing Bcl-2 and Bcl-X_L_ | *in vitro* | Hsu et al., 2018 |
| Regulating metabolic activity | Hormone replacement | ERβ-selective transcriptional activity and recruitment of coregulators | *in vitro* | An et al., 2001 |
|  | Preventive effects in diabetic complications | Inhibiting the glycosylation of albumin | *in vitro* | Asgary et al., 2002 |
|  | Inhibitory activity on fatty acid amide hydrolase | Inhibiting fatty acid amide hydrolase in a mixed-type manner | *in vitro* and *in vivo* | Thors et al., 2010 |
|  | Skin-whitening effect | Inhibitory activity on melanogenesis | *in vitro* and *in vivo* | Lin et al., 2011 |
|  | Antihyperglycemic effect | Restoring plasma glucose, insulin, glycosylated hemoglobin and the activities of carbohydrate metabolic enzymes | *in vivo* | Harini et al., 2012 |
|  | Alternative to hormone therapy | Serotonergic mechanisms | *in vivo* | Hellström and Muntzing, 2012 |
|  | Preventive effect on bone loss | Regulating the growth and activity of osteoblasts and osteoclasts | *in vivo* | Su et al., 2013 |
|  | Hypoglycemic and antilipemic activities | Repairing pancreatic beta cells | *in vivo* | Azizi et al., 2014 |
|  | Protective effects on articular cartilage | Attenuating the IL-1β-induced activation of NF-κB | *in vitro* and *in vivo* | Wu et al., 2014 |
|  | Gastroprotective effects | Enhancing cellular metabolic cycles by increasing SOD, NO, and Hsp70 and decreasing MDA and Bax | *in vivo* | Hajrezaie et al., 2015 |
|  | Attenuation of neuropathic pain | Majorly alleviating mechanical allodynia and moderately alleviating mechanical hyperalgesia | *in vivo* | Chundi et al., 2016 |
|  | Improving hepatic steatosis and insulin resistance | Regulating hepatic lipid by activating PPAR‐α and glucose metabolic pathways | *in vivo* | Park et al., 2016 |
|  | Ameliorating sugar induced cataractogenesis | Inhibiting glycation, glycation induced lens opacity, advanced glycation end products, aldose reductase and lens protein aggregation | *in vitro* | Patil et al., 2016 |
|  | Antihypertensive effect | eNOS dependent pathway | *in vitro* and *in vivo* | Sachdeva et al., 2016 |
|  | Linking to sex hormone-dependent disorders | Upregulating ERβ expression | *in vivo* | Elsherbini et al., 2017 |
|  | Hypolipidemic effect | Preventing the formation of ox-LDL by activating the expression of enzymes with antioxidant activity, inhibiting foam cell formation by promoting cholesterol efflux, and keeping the transformation of different lipoproteins normal by regulating relevant enzyme activity | *in vitro* and *in vivo* | Xue et al., 2017 |
|  | Alternative estrogen therapy | Decreasing the serum levels of urea, creatinine, and uric acid, downregulating renal TNF-α and iNOS expression and upregulating cutaneous TGF-β expression | *in vivo* | Galal et al., 2018 |
|  | Improving diabetic retinopathy | Reducing VEGF by inhibiting NF-κB activation | *in vivo* | Mehrabadi et al., 2018 |
|  | Improving bone health | Ameliorating the adverse bone health decrements caused by adjuvant anastrozole | *in vivo* | Mohamed et al., 2018 |
|  | Reducing insulin resistance and improving insulin sensitivity | Increasing the expression of SIRT1 | *in vivo* | Oza and Kulkarni, 2018 |
| Anti-proinflammatory activity | In macrophage and monocytes | Reducing the synthesis of prostaglandin E2 and/or thromboxane B2 and inhibiting COX activity | *in vitro* | Lam et al., 2004 |
|  | In osteoblastic cells | Inhibiting the H_2_O_2_-induced production of inflammatory mediators | *in vitro* | Lee and Choi, 2005 |
|  | In T cells | Upregulating interleukin-4 production via the PI3-K/PKC/NF-AT and PKC/p38 MAPK/AP-1 pathways | *in vitro* | Park et al., 2006 |
|  | In fibroblasts, embryonic kidney cells, and breast cancer cells | Inhibiting NF-κB–driven gene expression by attenuating the ERK-MAPK/MSK1 cascade and interfering with MSK1 kinase activity and histone acetyltransferase/histone deacetylase cofactor activities | *in vitro* | Vanden et al., 2006 |
|  | In lipopolysaccharide-stimulated macrophages | Activating PPARα, reducing proinflammatory cytokines, increasing anti-inflammatory cytokines, and/or inhibiting NF-κB, iNOS and COX2 | *in vitro* | Mueller et al., 2010 |
|  | Treatment of allergic asthma and chronic obstructive pulmonary disease | Suppressing airway hyperresponsiveness, recruiting inflammatory cells, and enhancing cytokine secretion, IgG, and IgE | *in vivo* | Ko et al., 2011 |
|  | Preventing proliferation and inflammation in cancer cells | Inhibiting iNOS expression and p38-MAPK and ATF-2 phosphorylation and blocking NF-κB nuclear translocation | *in vitro* | Kole et al., 2011 |
|  | In macrophages | PPARγ-dependent pathway | *in vitro* and *in vivo* | Qiu et al., 2012 |
|  | Protecting against acute carbon tetrachloride-induced hepatotoxicity | Inhibiting the expression of iNOS and COX2 and the secretion of proinflammatory cytokines and moderating the immune response | *in vivo* | Breikaa et al., 2013 |
|  | Anti-allergic effects | Suppressing the antigen-induced phosphorylation of the downstream signaling intermediates (MAPK/Akt) and attenuating the production of IgE-mediated proinflammatory cytokines | *in vitro* | Chung et al., 2013 |
|  | Anti-sUV-induced skin inflammation | Suppressing sUV-induced COX-2 expression mediated through MLK3 inhibition | *in vitro* | Lim et al., 2013 |
|  | In umbilical vein endothelial cells | Suppressing VCAM-1, ICAM-1, and E-selectin expression induced by LPS and activating PPAR-γ, thereby attenuating NF-κB activation | *in vitro* | Ming et al., 2015 |
|  | In LPS-stimulated microglia | Activating PPAR-γ, thereby attenuating NF-κB activation and the release of proinflammatory mediators | *in vitro* | Zhang and Chen, 2015 |
|  | In chondrocytes and articular cartilage | Suppressing IL-1β-induced inflammatory mediators (iNOS and COX-2) and inflammatory cytokines (TNFα, IL-6, IL-1α, IL-1β, IFNγ, IL-2 and GM-CSF) and suppressing the phosphorylation of NF-κB | *in vitro* | Oh et al., 2016 |
| Anti-infection | Protection against human herpesvirus 6 infection | Affecting viral attachment and internalization by inhibiting tyrosine kinase | *in vitro* | Cirone et al., 1996 |
|  | Selective inhibitory effect against intestinal tract colonizing bacteria | Unknown (insufficient data) | *in vitro* | Sklenickova et al., 2010 |
|  | Anti-inflammation in H5N1 influenza A virus-infected cells | Suppressing virus-induced activation of AKT, ERK 1/2, and NF-kB and inhibiting virus-induced production of IL-6, IL-8, and IP-10 | *in vitro* | Sithisarn et al., 2013 |
|  | Inhibitory activity on *Chlamydia* | Suppressing inclusion counts and decreasing the mean bacterial inclusion size | *in vitro* | Hanski et al., 2014 |
|  | Anti-H5N1 influenza A virus infection | Reducing viral replication by inhibiting the H5N1-induced activation of AKT, ERK1/2, and NF-κB and enhancing H5N1-induced ROS formation | *in vitro* | Michaelis et al., 2014 |
|  | Effect on enterovirus 71 infection | Without anti-enterovirus 71 activity because of its hydroxyl group at the C5 position | *in vitro* | Li et al., 2017 |
|  | Anti-*Salmonella* infection | Activating AMPK/ULK1/mTOR-mediated autophagy and inducing macrophage extracellular traps | *in vitro* and *in vivo* | Zhao et al., 2018 |
| Neuroprotective activity | Treatment of neurodegenerative diseases including Parkinson's disease | Protecting dopaminergic neurons against LPS-induced neurotoxicity associated with the inhibition of microglia activation and proflammatory factor generation | *in vitro* and *in vivo* | Chen et al., 2007 |
|  | Protection against H2O2-induced cell death in a human cortical cell line | Associated with its antioxidant activity | *in vitro* | Occhiuto et al., 2009 |
|  | Protection against glutamate-induced cytotoxicity | Inhibiting apoptosis by reducing caspase-3 activity | *in vitro* | Tan et al., 2013 |
|  | Protection against Alzheimer's disease | Elevating acetylcholinesterase indirectly by decreasing cholinesterase activity and improving memory. Antioxidant effect by reducing lipid peroxidation and increasing the glutathione levels | *in vivo* | Biradar et al., 2014 |
|  | Protection against Alzheimer's disease | Binding the preformed fibril structure of β-amyloid_25–35_ | *in vitro* | Ghobeh et al., 2014 |
|  | Protection against focal cerebral ischemia/reperfusion | Inhibiting p38-mediated inflammatory responses | *in vivo* | Wang et al., 2015 |
|  | Attenuating proinflammatory responses in microglia | Inhibiting ROS, NO production and iNOS expression and downregulating the production of TNF-α and IL-1β at the transcriptional level in LPS-stimulated microglial cells via the MAPK (JNK, ERK, and p38) pathway | *in vitro* | Wu et al., 2015 |
|  | Protection against Alzheimer's disease | Inhibiting β-amyloid_25–35_-induced apoptosis by suppressing caspase activity | *in vitro* | Tan and Kim, 2016 |
|  | Protection against lipopolysaccharide-induced damage to dopaminergic neurons | Decreasing the levels of IL-1β, IL-6 and TNF-α, inhibiting the phosphorylation of MAPK (ERK, JNK, and p38), and inhibiting ROS production | *in vitro* and *in vivo* | Wang et al., 2016 |
|  | Protection against Alzheimer's disease | Inhibiting a mitochondrial-dependent apoptosis pathway but also by binding the allosteric site of β-site amyloid precursor protein cleaving enzyme 1 | *in vitro* | Youn et al., 2016 |
|  | Protection against stroke | Inducing glutamate oxaloacetate transaminase expression | *in vitro* and *in vivo* | Khanna et al., 2017 |
|  | Dopaminergic neuroprotection | Maintaining redox balance | *in vivo* | Yu et al., 2017 |
|  | Protection against Parkinson’s and Alzheimer’s diseases | Inhibiting monoamine oxidase-B (MAO-B) via hydrophobic interactions between BCA and MAO-B | *in vitro* | Zarmouh et al., 2017 |
|  | Neuroprotective activity post subarachnoid hemorrhage | Reducing inflammatory injury and neuronal apoptosis and suppressing the TLRs/TIRAP/MyD88/NF-κB pathway | *in vivo* | Wu et al., 2018 |
