# Supplementary material for: Perspectives Regarding the Role of Biochanin A in Humans
Source: Front Pharmacol. 2019 Jul 12;10:793. doi: 10.3389/fphar.2019.00793 (PMC6639423; doi:10.3389/fphar.2019.00793)
Supplement: Supplementary file 2 [file Table_2.docx]

**TABLE S2** Compilation of the pharmacokinetic data on BCA: drug-drug interactions

| **Drug** | **Mechanisms** | **Effects** | **References** |
| --- | --- | --- | --- |
| daunomycin | Inhibits P-gp-mediated cellular efflux | Increases sensitivity to the drug; potentiates drug cytotoxicity in cancer cells | Zhang et al., 2003; Chung et al., 2005;  Zhang et al., 2010 |
|  | Inhibits MRP1-mediated drug transport |  | Versantvoort et al., 1993; Nguyen et al., 2003; |
| doxorubicin | Inhibits P-gp-mediated cellular efflux | Increases sensitivity to the drug; potentiates drug cytotoxicity in cancer cells | Zhang et al., 2003; Dash and Konkimalla, 2017 |
| vinblastine | Inhibits MRP1-mediated drug transport |  | Nguyen et al., 2003 |
| mitoxantrone | Stimulates P-gp-mediated cellular efflux and inhibits BCRP-mediated cellular efflux | Decreases the oral bioavailability of the drug | An and Morris, 2010 |
| quinolones | Suppresses the norA protein and the efflux system (ATP-binding ABC transporters) | Increases drug accumulation and decreases its MIC | Liu et al., 2011; Zou et al., 2014; Jin et al., 2017; Cannalire et al., 2017 |
| paclitaxel | Inhibits P-gp-mediated cellular efflux | Increases the oral bioavailability of the drug | Peng et al., 2006 |
| digoxin | Inhibits P-gp-mediated cellular efflux | Increases the oral bioavailability of the drug | Peng et al., 2006 |
| fexofenadine | Inhibits Oatp3 in the intestine | Decreases the oral bioavailability of the drug | Peng et al., 2006 |
| tamoxifen | Unclear but may stimulate P-gp-mediated cellular efflux | Decreases the oral bioavailability of the drug | Singh et al., 2012 |
| arsenic | Antioxidant activity | Protects heart and kidney tissue | Jalaludeen et al., 2015 |
| saquinavir | Stimulates P-gp-mediated cellular efflux | Decreases the oral bioavailability of the drug and alters its pharmacokinetic profile | Li et al., 2016 |
| sorafenib | Enhances the antiproliferative and apoptotic effects of sorafenib | Synergistic cytotoxicity in cancer cells | Youssef et al., 2016 |
| cisplatin | Anti-inflammatory and antiapoptotic activities | Nephroprotective effects | Suliman et al., 2018 |
